# Post-renal Biopsy Retroperitoneal Haematoma Accompanied by Decreased Coagulation Factor XIII Levels in Immunoglobulin A Nephropathy

**DOI:** 10.7759/cureus.54026

**Published:** 2024-02-11

**Authors:** Yoko Hirano, Shoichiro Kanda, Moe Hidaka, Motohiro Kato, Masako Nishikawa, Yutaka Yatomi, Hiroyuki Tanaka, Akiko Kinumaki, Yuko Kajiho, Yutaka Harita

**Affiliations:** 1 Department of Pediatrics, The University of Tokyo, Tokyo, JPN; 2 Department of Clinical Laboratory Medicine, The University of Tokyo, Tokyo, JPN

**Keywords:** pre-renal biopsy evaluation, haematoma, iga nephropathy, factor xiii, biopsy complications

## Abstract

Post-biopsy bleeding is the primary complication of renal biopsy. Retroperitoneal haematoma is a rare but severe bleeding complication; it commonly occurs among patients who have risk factors or vascular lesions. The bleeding risks in patients with immunoglobulin A (IgA) nephropathy (IgAN) have been discussed in the literature, but clinical data are lacking. Here, we report a case of a post-biopsy retroperitoneal haematoma accompanied by decreased coagulation factor XIII (FXIII) in a patient with IgAN. A 14-year-old male patient with haematuria and proteinuria but no bleeding or family history of bleeding underwent pre-renal biopsy evaluation that showed no coagulation abnormalities. He underwent percutaneous renal biopsy, and the histopathological diagnosis was IgAN. Five days after the biopsy, he presented with delayed bleeding from a retroperitoneal haematoma. During the workup for undiagnosed haemorrhagic diatheses, a mildly decreased FXIII level was discovered. This result suggested the possibility of bleeding complications associated with decreased FXIII. Some bleeding diatheses, including FXIII deficiency, cannot be evaluated in routine pre-biopsy coagulation tests. Mild FXIII deficiency can increase the risk of post-biopsy bleeding complications. Therefore, physicians should consider unevaluated haemorrhagic diatheses when a patient presents with major bleeding complications or delayed bleeding following renal biopsy without any known risk factors or vascular lesions.

## Introduction

Post-biopsy bleeding is the primary complication of renal biopsies. Notably, a retroperitoneal haematoma is an uncommon but severe bleeding complication with a nonspecific presentation, such as abdominal or back pain and symptoms of hypovolaemia [1,2]. It usually occurs in patients with risk factors or vascular lesions, including coagulation disorders [3], arterial injuries [1], and pseudoaneurysms [2]. Most bleeding diatheses are detected during pre-biopsy evaluations. Additionally, various risk factors for renal biopsy complications have been studied, and a histopathological type is one of them. For example, a previous report indicated that patients with immunoglobulin A (IgA) nephropathy (IgAN) had a high risk of major bleeding complications related to renal biopsies [4]. However, other studies suggested contrary results [5]. Thus, the risk of bleeding complications in patients with IgAN is still under discussion.

Here, we report a case of post-renal biopsy retroperitoneal haematoma accompanied by decreased coagulation factor XIII (FXIII) levels in a patient with IgAN.

This article was previously presented as an oral presentation at the 58th Annual Meeting of the Japanese Society for Pediatric Nephrology on July 1, 2023.

## Case presentation

A 13-year-old Japanese boy underwent urinary screening in school and was found to have haematuria and proteinuria. He underwent an ultrasound-guided percutaneous renal biopsy at 14 years of age. The patient had no bleeding history. He also had no family history of bleeding or consanguineous marriages. On admission, he had unremarkable vital signs, a height of 177.4 cm, and a weight of 72.3 kg (BMI, 23.0 kg/m^2^). Physical examination revealed no significant abnormalities. Laboratory analysis results were unremarkable, including coagulation tests (Table 1). Urinalysis showed macroscopic haematuria and proteinuria.

**Table 1:** Laboratory tests on first admission (pre-biopsy) APTT, activated partial thromboplastin time; Cr, creatinine; eGFR, estimated glomerular filtration rate; FDP, fibrinogen/fibrin degradation products; IgA, immunoglobulin A; IgG, immunoglobulin G; IgM, immunoglobulin M; PT, prothrombin time; INR, international normalized ratio.

Parameters	Value	Parameters	Value
Complete blood count		Serum chemistry	
White-cell count (per μL)	8,900	Total protein (g/dL)	6.7
Red-cell count (per μL)	4,920,000	Albumin (g/dL)	4.2
Haemoglobin (g/dL)	14.6	IgA (mg/dL)	300
Haematocrit (%)	43.4	IgG (mg/dL)	964
Platelet count (per μL)	265,000	IgM (mg/dL)	70
Coagulation tests		Sodium (mmol/litre)	143
PT (sec)	11.2	Potassium (mmol/litre)	3.9
PT-INR	0.97	Chloride (mmol/litre)	106
APTT (sec)	24.3	Calcium (mg/dL)	9.1
Fibrinogen (mg/dL)	268	Inorganic phosphorus (mg/dL)	4.3
FDP (μg/mL)	2.5	Uric acid (mg/dL)	5.4
D-dimer (μg/mL)	0.5	Creatine kinase (U/litre)	96
Bleeding time (min)	1	Urea nitrogen (mg/dL)	11.4
Urinalysis		Creatinine (mg/dL)	0.61
Specific gravity	1.023	C-reactive protein (mg/dL)	0.04
Ketones	Negative	Lactate dehydrogenase (U/litre)	153
Nitrite	Negative	Alanine aminotransferase (U/litre)	25
Blood	3+	Aspartate aminotransferase (U/litre)	18
Protein	2+	Alkaline phosphatase (U/litre)	147
Protein/Creatinine ratio (g/gCr)	0.57	Total bilirubin (mg/dL)	0.5
Red cells (per high-power field)	50–99	eGFR (mL/min/1.73m^2^)	143
White cells (per high-power field)	<1		

Left renal biopsy was performed using a 16-gauge automated needle under intravenous sedation. The histopathological diagnosis was IgAN (Oxford Classification/MEST-C (mesangial hypercellularity (M), endocapillary hypercellularity (E), segmental glomerulosclerosis (S), tubular atrophy/interstitial fibrosis (T), and crescents (C)) score of M1/E0/S1/T0/C1). After the biopsy, ultrasonography showed a perinephric haematoma measuring 0.8 × 2.8 cm.

**Figure 1:**
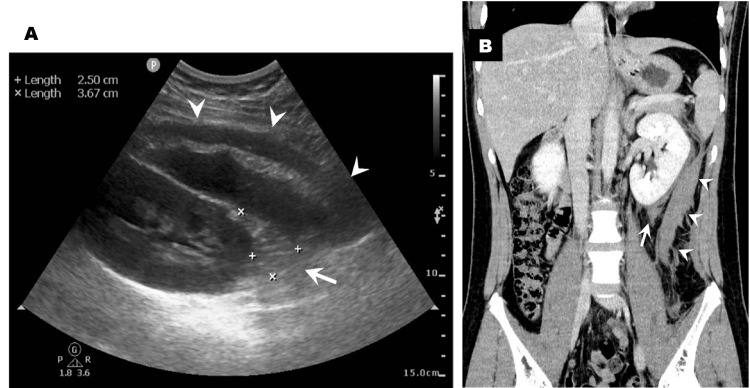
Imaging findings of the patient after renal biopsy. (A) Dorsal view of renal ultrasonography shows retroperitoneal (arrowheads) and perinephric haematomas (arrow). The layer of mixed echogenicity suggests repeated haemorrhage. (B) Abdominal contrast-enhanced computed tomography shows retroperitoneal (arrowheads) and perinephric (arrow) haematomas. The retroperitoneal haematoma (measuring 3 × 7 × 12 cm) extends from the left kidney to the left iliopsoas muscle. Extravasation of contrast media, pseudoaneurysm, or arteriovenous fistula was not detected.

**Figure 2:**
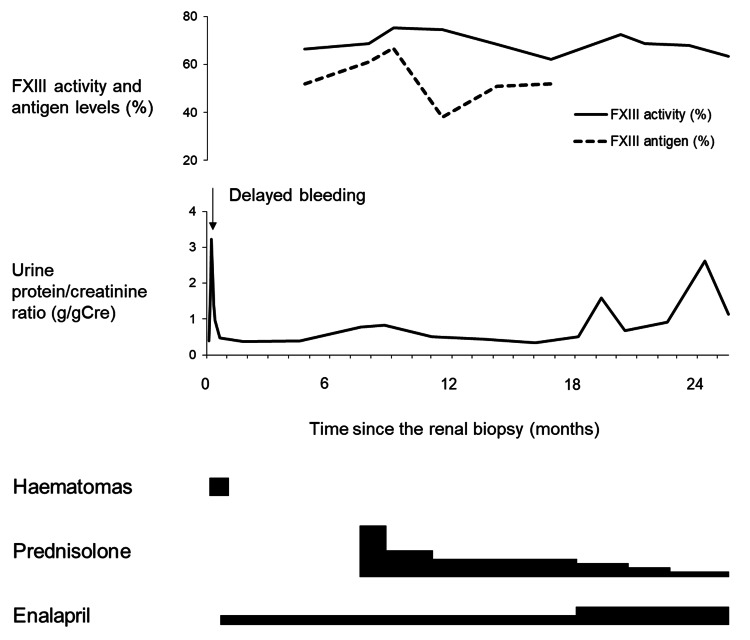
Clinical course of the patient after renal biopsy. The urine protein/creatinine ratio temporarily increased after the delayed bleeding but rapidly recovered to the baseline. The haematomas disappeared one month after the delayed bleeding. Four months later, decreased FXIII levels were discovered during detailed investigations. As treatment for IgA nephropathy, the patient was started on enalapril after discharge and prednisolone seven months later. The dose of prednisolone was gradually reduced and finally stopped. The FXIII activity levels normalized eight months later, and the FXIII activity and antigen levels thereafter remained in the normal to mildly deficient range. The FXIII activity/antigen remained at >1.0. The correlation between the urine protein/creatinine ratio and FXIII levels was unclear. FXIII, coagulation factor XIII; IgA, immunoglobulin A

The day after the biopsy, the patient reported left periumbilical and back pain. His haemoglobin level was 13.4 g/dL, and the perinephric haematoma had not changed significantly. His pain was immediately relieved by pentazocine, and he was discharged home five days after the biopsy. However, he returned to our hospital the day after discharge because of sudden-onset fever, left back pain, and gross haematuria. He appeared unwell, and laboratory investigations showed leucocytosis (12,700/μL), an elevated C-reactive protein level (6.43 mg/dL), and a decreased haemoglobin level (12.7 g/dL). Urinalysis showed gross haematuria and worsened proteinuria. In addition to the enlargement of the perinephric haematoma, which now measured 2.5 × 3.7 cm, a retroperitoneal haematoma measuring 3 × 7 × 12 cm was found by ultrasonography and contrast-enhanced computed tomography (Figures 1A, 1B). There was no extravasation of contrast media, no pseudoaneurysm, and no arteriovenous fistula. The haemoglobin level had decreased to 11.8 g/dL, but the patient did not need blood transfusion or surgical/radiological intervention. Urine and blood cultures were negative, and the fever was suggested to be a haematoma absorption fever. Almost all the urinary red blood cells were dysmorphic, which indicated that the gross haematuria might have been a result of IgA-related glomerular inflammation. The symptoms disappeared with bed rest, and he was discharged home 12 days after the biopsy. The haematomas disappeared one month after the delayed bleeding. As a treatment for IgAN, we started the patient on enalapril and added prednisolone seven months later (Figure 2).

Detailed laboratory investigations were performed to evaluate the patient for a bleeding disorder; these were not part of the pre-biopsy testing (Table 2). As a result, decreased FXIII levels were revealed (activity, 66.4% (normal range, 70-140%), antigen, 52% (normal range, ≥70%)). The detailed investigations revealed no other abnormalities. The FXIII activity normalized eight months later without replacement therapy (Figure 2), but the FXIII antigen level remained below 70%. Thereafter, the FXIII levels remained within the normal to mildly deficient range (activity, 66.4-75.7%; antigen, 38-67%; activity/antigen ratio, 1.1-2.0), and the patient had no symptoms on follow-up at 16 years of age.

**Table 2:** Detailed laboratory studies for haemorrhagic diatheses and underlying diseases (four months after biopsy) Ab, antibody; ANA, antinuclear antibodies; APTT, activated partial thromboplastin time; CL-GPI, cardiolipin β2-glycoprotein Ⅰ; FDP, fibrinogen/fibrin degradation products; IgG, immunoglobulin G; PAI-1, plasminogen activator inhibitor 1; PIC, α2-plasmin inhibitor–plasmin complex; PT, prothrombin time; PT-INR, prothrombin time–international normalized ratio; TAT, thrombin–antithrombin complex; vWF, von Willebrand factor.

Parameters	Value	Reference Range
White-cell count (per μL)	8,700	4,500–13,000
Red-cell count (per μL)	5,140,000	4,250,000–5,600,000
Haemoglobin (g/dL)	15.1	12.6–16.5
Haematocrit (%)	43.6	36.4–48.0
Platelet count (per μL)	280,000	170,000–410,000
PT (sec)	10.7	10–12
PT-INR	0.93	1.0
APTT (sec)	24.8	24–34
Lupus anticoagulant (index)	1.2	0–1.2
Anti-cardiolipin IgG (U/mL)	4	0–9
Anti-CL-GPⅠ Ab (U/mL)	1.3	0–3.4
ANA (index)	Negative	<1:40
Thrombomodulin (FU/mL)	3.2	2.1–4.1
Fibrinogen (mg/dL)	314	168–355
FDP (μg/mL)	2.5	≤5
D-dimer (μg/mL)	0.5	≤1.0
Antithrombin activity (%)	112	80–130
Antithrombin antigen (mg/dL)	31	23.6–33.5
TAT (ng/mL)	1.2	0–3.9
PIC (μg/mL)	0.8	0–0.7
Plasminogen (%)	131	80–130
α_2_ antiplasmin (%)	126	80–130
Total PAI-1 (ng/mL)	20	0–50
vWF activity (%)	171	50–150
vWF antigen (%)	197.2	50–155
Factor VIII activity (%)	198.4	60–140
Factor IX activity (%)	138.8	60–140
Factor XIII activity (%)	66.2	70–140
Factor XIII antigen (%)	52	≥70
Platelet aggregation test	Normal	Normal

## Discussion

This case revealed two critical clinical issues. First, an unrecognized haemorrhagic diathesis can lead to severe bleeding complications following renal biopsy. Generally, the platelet count, prothrombin time, activated partial thromboplastin time, fibrinogen, and fibrinogen/fibrin degradation products (or D-dimer) are examined in pre-biopsy screening [6]. However, patients with platelet function disorders, some fibrinolysis disorders, and coagulation disorders (including FXIII deficiency (FXIIID)) have no abnormalities in routine coagulation screening. Additionally, some haemorrhagic diatheses are routinely asymptomatic. Not all patients have bleeding and family histories, even if the disorder is congenital [7]. Our patient initially seemed to have no risk factors, but he developed severe bleeding complications, and decreased FXIII levels were eventually revealed during detailed investigations. Therefore, if a patient presents with severe bleeding complications without any vascular lesions or risk factors for renal biopsy complications, physicians should consider unevaluated haemorrhagic diatheses regardless of the bleeding or family history.

Second, mild FXIIID can be associated with post-biopsy haemorrhage. FXIII is a crucial enzyme in blood clot stabilization. FXIII also has a range of other functions, including wound healing, angiogenesis, and pregnancy maintenance [8]. FXIIID may be congenital or acquired, and congenital FXIIID is a rare autosomal recessive bleeding disorder [9]. FXIII activity in patients with severe congenital FXIIID is undetectable [9], while in heterozygous FXIIID, FXIII activity is typically between 30% and 70% [10]. The FXIII activity level required to prevent spontaneous major bleeding is 15% [11], and the requirement for FXIII increases during surgery and bleeding because of hyperconsumption. Patients with heterozygous FXIIID are usually asymptomatic, but some develop severe bleeding complications under special conditions such as surgery, trauma, and pregnancy.

Postoperative/tooth extraction bleeding has been reported in patients with heterozygous FXIIID who had FXIII activity between 23% and 71% [10]. The bleeding risk after surgical procedures is higher in heterozygotes with FXIIID than in healthy individuals because the baseline levels of FXIII are lower [10]. By contrast, acquired FXIIID is caused by the generation of antibodies, hyperconsumption, or hyposynthesis due to an underlying comorbidity [9]. Recurrent postoperative haematoma was reported in a patient with mild acquired FXIIID (FXIII activity of 69%)[12]. Recurrent post-tonsillectomy haemorrhage may be associated with mild FXIIID (FXIII activity of 48%-58%) [13]. Therefore, these reports suggest that mildly decreased FXIII levels can lead to recurrent bleeding after surgical procedures, regardless of whether FXIIID is congenital or acquired. In the present case, the patient’s FXIII levels were mildly decreased. He also presented with delayed bleeding, which is a characteristic finding in FXIIID. There were no other abnormalities in platelet function, fibrinolysis, or coagulation factors. FXIIID secondary to bleeding was not suggested because the patient’s FXIII levels were within the mild deficiency range even one year after the biopsy. Therefore, the possibility of atypical acquired FXIIID or heterozygous FXIIID remains. We could not obtain critical samples in the acute phase; however, considering the patient’s clinical course, the FXIII levels had probably decreased in the peri-biopsy period. As described above, slight FXIIID can lead to severe bleeding complications after surgical procedures. These observations suggest that decreased FXIII levels can be associated with haematoma enlargement and delayed bleeding, as in the present case. Mildly decreased FXIII levels are asymptomatic under ordinary conditions, but they can increase the risk of bleeding complications after renal biopsy. Therefore, physicians should suspect FXIIID when patients present with atypical or severe haemorrhagic complications, particularly delayed bleeding. Although most major bleeding complications of renal biopsy occur within 24 hours of the procedure [14], some haemorrhagic diatheses are characterized by delayed bleeding. Physicians should also pay attention to the possibility of major bleeding complications after discharge.

The pathological diagnosis in our patient was IgAN. IgA vasculitis (IgAV), which has pathogenetic and histopathologic features similar to those of IgAN, is one of the underlying diseases of acquired FXIIID [9]. In patients with IgAV, FXIIID is associated with gastrointestinal bleeding complications, and the mechanisms of FXIIID are hypothesized to be hyperconsumption as well as degradation by proteases [15]. Furthermore, various coagulation abnormalities have recently been reported in IgAN and IgAV, including a shortened prothrombin time and activated partial thromboplastin time [16] and an elevated von Willebrand factor level [17,18]. Most laboratory abnormalities suggest hypercoagulation because of endovascular injuries and are associated with the severity or activity of diseases. Additionally, with respect to the immunological characteristics, anti-tissue transglutaminase antibodies are detected in some patients with IgAN and IgAV [19]. FXIII is a transglutaminase family protein, and anti-FXIII antibodies are observed in some patients with immunological diseases [20]. Therefore, although we could not confirm the presence of anti-FXIII antibodies in our patient’s serum, it is possible that an immunological mechanism was involved in the decrease in FXIII in this patient with IgAN. Peters et al. reported that patients with IgAN have an increased risk of major bleeding complications and hypothesized that FXIII is involved [4]. On the other hand, both IgAN and FXIIID can develop due to various causes, including genetic factors, environmental factors, and immune reactions. Therefore, these two conditions could be coincidental due to their respective causes or their autoimmune or idiopathic origin. Therefore the relationship between IgAN and FXIIID is unclear.

## Conclusions

This report described a patient with IgAN presenting with renal biopsy-related severe bleeding complications accompanied by decreased FXIII levels. Physicians should consider unevaluated haemorrhagic diatheses if a patient presents with severe renal complications or delayed bleeding without any known risk factors or vascular lesions. Undiagnosed haemorrhagic diathesis could be hidden in cases of unexplained post-biopsy bleeding complications.
